# Integrated Network Pharmacology and Experimental Validation Approach to Investigate the Therapeutic Effects of Capsaicin on Lipopolysaccharide-Induced Acute Lung Injury

**DOI:** 10.1155/2022/9272896

**Published:** 2022-01-30

**Authors:** Peihui Liu, Jindou Hao, Jie Zhao, Rong Zou, Juan Han, Jia Tian, Wanqu Liu, Hao Wang

**Affiliations:** ^1^Department of Pediatrics, Affiliated Shenzhen Maternity & Child Healthcare Hospital, Southern Medical University, Shenzhen, Guangdong, China; ^2^Department of Neonatology, Affiliated Shenzhen Maternity & Child Healthcare Hospital, Southern Medical University, Shenzhen, Guangdong, China; ^3^Department of Pharmacy, Affiliated Shenzhen Maternity & Child Healthcare Hospital, Southern Medical University, Shenzhen, Guangdong, China; ^4^Affiliated Shenzhen Maternity & Child Healthcare Hospital, Southern Medical University, Shenzhen, Guangdong, China

## Abstract

An integrated method combining network pharmacology and in vivo experiment was performed to investigate the therapeutic mechanism of capsaicin (Cap) against acute lung injury. The potential key genes and signaling pathways involved in the therapeutic effect of Cap were predicted by the network pharmacology analyses. Additionally, the histological assessment, ELISA, and RT-qPCR were performed to confirm the therapeutic effect and the potential mechanism action involved. Our findings showed that TNF, IL-6, CXCL1, CXCL2, and CXCL10 were part of the top 50 genes. Enrichment analysis revealed that those potential genes were enriched in the TNF signaling pathway and IL-17 signaling pathway. *In vivo* experiment results showed that Cap alleviated histopathological changes, decreased inflammatory infiltrated cells and inflammatory cytokines, and improved antioxidative enzyme activities in the bronchoalveolar lavage fluid (BALF). Furthermore, Cap treatment effectively downregulated TNF, IL-6, NF-*κ*B, CXCL1, CXCL2, and CXCL10 in lung tissue. Thus, our findings demonstrated that Cap has the therapeutic effect on LPS-induced acute lung injury in neonatal rats via suppression of the TNF signaling pathway and IL-17 signaling pathway.

## 1. Introduction

Acute lung injury is the initial stage of the entire pathological progression of acute respiratory distress syndrome. It is also a life-threatening inflammatory syndrome with high mortality and morbidity [1]. Acute respiratory distress syndrome is the more severe manifestation of acute lung injury, which is characterized by lung inflammation, pulmonary edema, diffuse alveolar damage, and increased neutrophil infiltration [2, 3]. Newborns, particularly preterm infants, are susceptible to acute lung injury, which is one of the most common causes of neonatal death [4, 5]. Besides, it has been reported that uncontrolled pulmonary inflammation is involved in the pathological process of acute lung injury in infants [6]. To date, current treatment strategies have not been effective in decreasing the mortality and incidence of acute lung injury [7]. Therefore, it is extremely urgent to develop an effective drug for the treatment of acute lung injury.

Lipopolysaccharide (LPS) is a pathogenic endotoxin of Gram-negative bacteria that promotes the generation of inflammatory cytokines and reactive oxygen species [8]. Previous reports have revealed that administration of bacterial LPS to neonatal rats could cause inflammatory injury of the neonatal lung [9, 10]. Therefore, LPS has been widely used as an inducer of acute lung injury in the establishment of lung injury models [11, 12]. Several signaling pathways are involved in the pathogenesis of LPS-induced acute lung injury, which is closely related to the regulation of oxidative stress and inflammatory response [8]. Accumulating reports revealed that LPS could trigger pulmonary inflammation via activation of nuclear factor-kappa B (NF-*κ*B), which leads to excessive proinflammatory cytokines such as IL-6 and TNF-*α* [13, 14]. Subsequently, these inflammatory cytokines could further cause endothelial and epithelial cell injury in the lung tissue. Thus, inhibiting pulmonary proinflammatory cytokine levels might be an effective therapeutic strategy for the prevention and treatment of neonatal acute lung injury.

Capsaicin (Cap) is an active ingredient of chili pepper, which has been widely investigated for its various pharmacological activities, including analgesic, anti-inflammatory, antioxidant, circadian modulatory, antilithogenic, cardioprotective, and anticarcinogenic effects [15, 16]. The previous reports indicated that Cap exerted the chemopreventive effects against lung carcinogenesis via decreasing the expression of inflammatory cytokines [17]. Cap pretreatment could decrease pulmonary arterial hypertension via inhibiting lung inflammation [18]. However, the therapeutic effect of Cap against LPS-induced acute lung injury has not yet been reported.

In the present study, the network pharmacology method was used to predict the potential key genes and signaling pathways of Cap in the treatment of acute lung injury. Besides, we established a newborn rat model of acute lung injury to further verify the therapeutic mechanism of Cap on acute lung injury.

## 2. Materials and Methods

### 2.1. Prediction of Target Genes of Cap

The keyword “capsaicin” was searched in the PubChem database (https://pubchem.ncbi.nlm.nih.gov/) to download 2D structure. Then, the potential genes of Cap were collected from the SwissTargetPrediction database (http://www.swisstargetprediction.ch/) and CTD database (http://ctdbase.org/).

### 2.2. Collection of Target Genes of Lung Injury

The keyword “lung injury” was searched in the CTD database (http://ctdbase.org/) and GeneCards database (http://www.genecards.org) to collect disease-related genes. Then, the duplicate genes between the two databases were integrated, and the collected genes were identified as lung injury-related genes. Finally, the intersection genes of Cap-related genes and lung injury-related genes were defined as potential therapeutic genes of Cap against lung injury.

### 2.3. Construction of the PPI Network

The common genes were imported into the STRING database (https://www.string-db.org/), the confidence score was set up as 0.9, and the species was set Homo sapiens. Then, the TSV format was downloaded and imported into Cytoscape software (3.8.0). The CytoHubba plugin of Cytoscape was used to screen the key genes of the network.

### 2.4. Animals

All animal experimental operations were strictly carried out in accordance with the guidelines approved by the Institutional Animal Care and Use Committee of Southern Medical University. Sprague-Dawley neonatal rats (10-16 g body weight and 4-8 days old) were obtained from the Animal Experimental Center of Guangdong Province and housed in a specific pathogen-free environment with standard conditions (55-60% humidity, 20-22°C temperature, and a 12 h dark/light cycle). All animals were allowed free access to water and food *ad libitum*.

### 2.5. Experimental Animal Model and Drug Interventions

All rats were randomly divided into a control group and four experimental groups (*n* = 8): lipopolysaccharide (LPS) group (intraperitoneal injection of 3 mg/kg of LPS to induce acute lung injury), LPS+2 mg/kg of Cap group, LPS+4 mg/kg of Cap group, and LPS+8 mg/kg of Cap group. After LPS exposure, the rats of the model group received subcutaneous injections of Cap daily for eight consecutive days. LPS (*Escherichia coli* 055:B5) was obtained from Sigma-Aldrich, and the dose selection was based on the previous report [10]. The doses of Cap used in the present study were according to the previous report and our preliminary experiment [19]. After the last Cap intervention for 24 h, the neonatal rats were sacrificed via intraperitoneal injection of pentobarbital (50 mg/kg). Lung tissues were collected and stored at -80°C before analysis.

### 2.6. Lung Tissue Histological Assessment

Lung tissues were fixed with 4% paraformaldehyde. The tissue samples were dehydrated using a series of concentrations of ethanol. The dehydrated samples were embedded in paraffin and then sliced into 5 *μ*m thick sections. Then, the sections were stained using a hematoxylin-eosin reagent and observed under a microscope.

### 2.7. Evaluation of Lung Edema

The collected lung tissues were rinsed with ice 0.9% saline and dried using filter paper. Then, the lung tissues were immediately weighed to obtain the wet weight. The lung tissues were dried by baking at 70°C for 48 h and weighed again to obtain the dry weight. Finally, the value of wet weight/dry weight was calculated to evaluate the lung edema.

### 2.8. Measurement of Inflammatory Infiltrated Cells and Inflammatory Cytokines in the Bronchoalveolar Lavage Fluid (BALF)

After the experiment, the BALF was prepared based on the previous study [20]. BALF was centrifuged at 1,500 g for 15 min at 4°C, and the supernatant was collected to obtain the infiltrated cells. Then, the sediment cells were resuspended with ice 0.9% saline and stained with Wright-Giemsa stain (Jiancheng Bioengineering Institute, China). Cells were counted using a hemocytometer under a microscope. The inflammatory cytokine (TNF-*α*, IL-6, and IL-1*β*) levels in BALF were measured using a commercial kit per the manufacturer's protocols (Jiancheng Bioengineering Institute, China).

### 2.9. Measurement of Protein Concentration, MPO Activity, and Antioxidative Enzyme Activities

The total protein level in the BALF was determined using a bicinchoninic acid protein kit based on the manufacturer's protocols (Jiancheng Bioengineering Institute, China). The lung tissue was homogenized in ice 0.9% saline and then centrifuged at 10000 g for 10 min at 4°C. The supernatant was collected, and the MPO activity and antioxidative enzyme activities (SOD and GSH) were measured using a commercial kit per the manufacturer's protocols (Jiancheng Bioengineering Institute, China).

### 2.10. Quantitative RT-PCR

Total RNA of lung tissue was extracted using TRIzol based on the manufacturer's instructions (TIANGEN, USA). 2 *μ*g of isolated RNA was reverse-transcribed into cDNA using a cDNA synthesis kit (Thermo Fisher, USA) based on the manufacturer's protocols. Then, the qPCR was carried out by using the SYBR GREEN Master Mix (Thermo Fisher, USA) in a LightCycler RT-PCR System (Roche Diagnostics, USA). The 2^–ΔΔCt^ method was used to quantify the mRNA expression levels. The primers are shown in Supplementary File Table S1.

### 2.11. Statistical Analysis

Values were expressed as mean ± standard deviation (SD). The GraphPad 5 software was used to perform the statistical analyses. Statistical significance of the difference between groups was measured by one-way ANOVA followed by Dunnett's multiple comparison test. A *P* value below 0.05 represents a significant difference.

## 3. Results

### 3.1. Identification of Potential Targets of Cap

**Figure 1:**
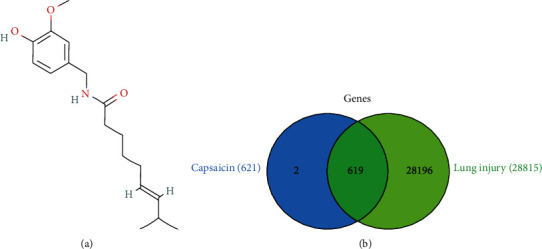
Potential genes of Cap against acute lung injury. Chemical structure of Cap (a). Venn map between target genes from Cap and lung injury (b).

The 2D chemical structure of Cap is presented in Figure 1(a). Based on SwissTargetPrediction and CTD databases, 621 potential genes of Cap were collected after the deletion of duplicate genes.

### 3.2. Identification and Analysis of Known Acute Lung Injury-Related Genes

**Figure 2:**
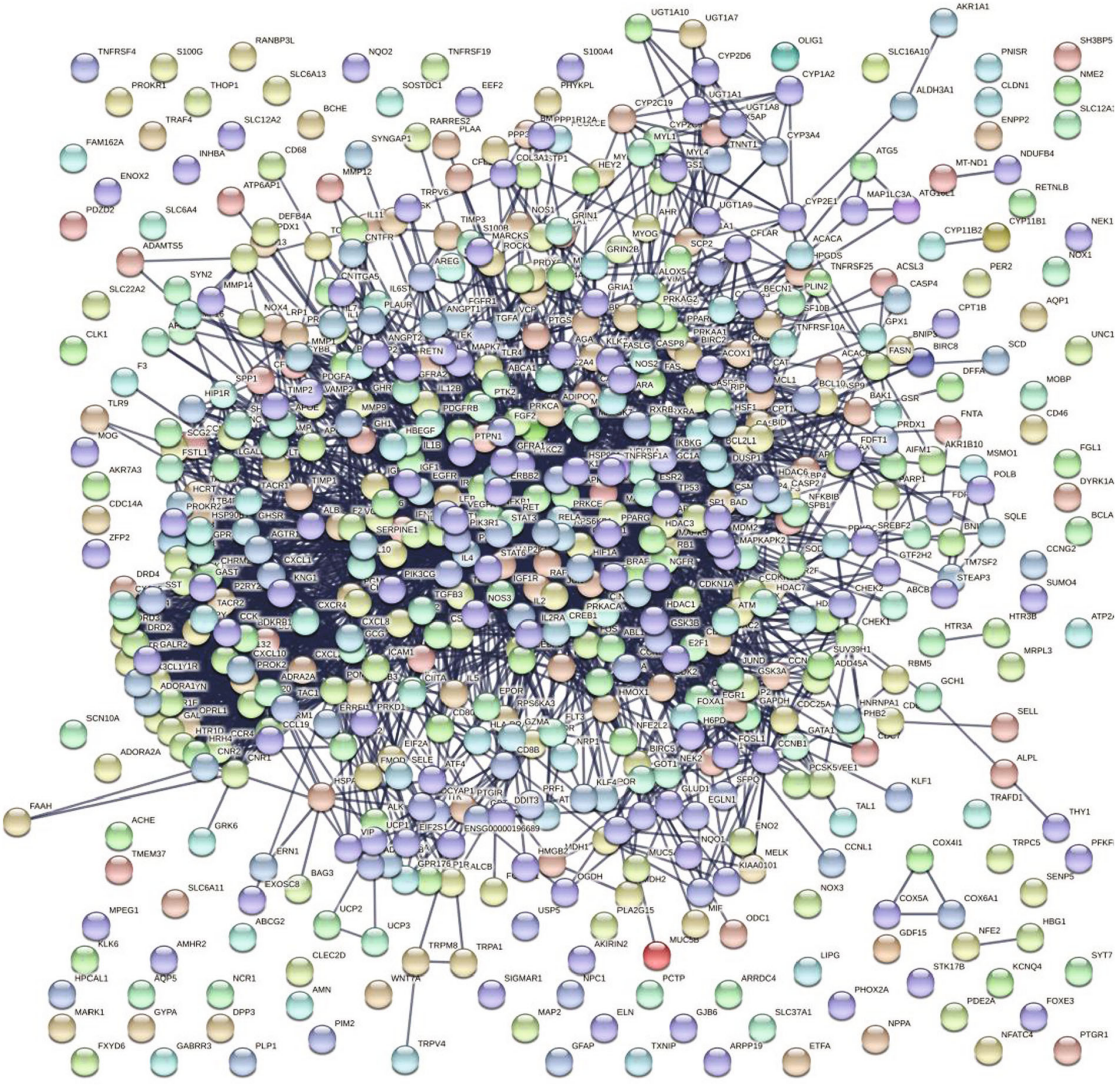
Construction of the PPI network for Cap against acute lung injury.

After the deletion of duplicate genes, a total of 28,815 genes were collected from CTD and GeneCards databases. Then, 621 potential genes from Cap were combined with 28,815 acute lung injury-related genes by using a Venn tool to identify the potential genes of Cap against acute lung injury. As shown in Figure 1(b), 619 common genes were identified and could represent the potential genes for acute lung injury treatment with Cap. As shown in Figure 2, the PPI network of the potential genes of Cap acting on acute lung injury was constructed by the STRING database.

### 3.3. Identification of Inflammation-Related Genes

**Figure 3:**
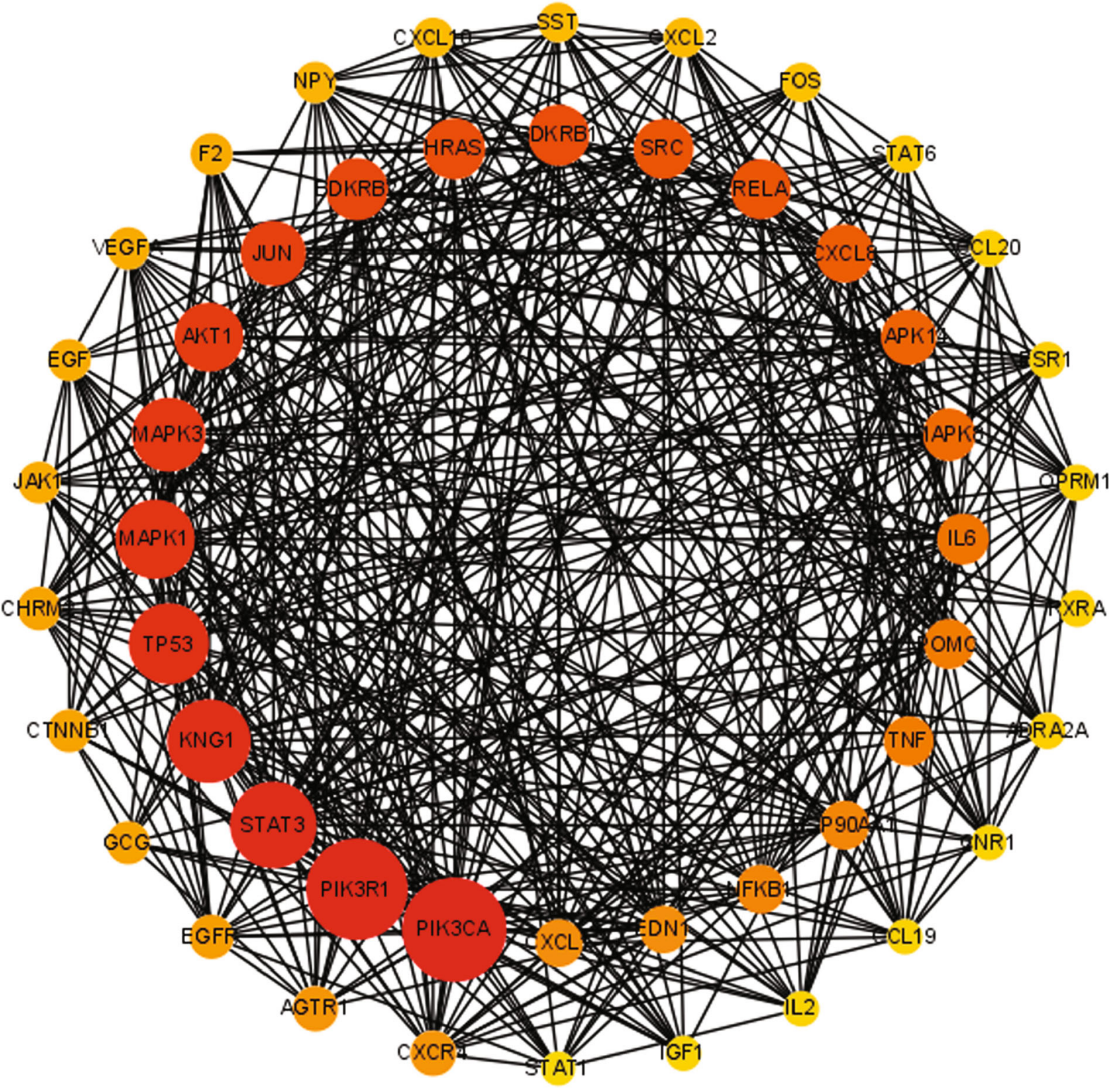
PPI network of top 50 potential genes. As the degree of the target genes increased, the color becomes darker and the circle becomes larger.

As shown in Figure 3, the top 50 genes were screened as key targets of Cap against acute lung injury. Most of them were inflammation-related genes, such as CXCL8, CXCR4, CXCL1, CXCL2, IL-6, CXCL10, CCL20, CCL19, IL-2, and TNF. The results showed that inflammatory response might be one of the mechanisms for Cap in the treatment of acute lung injury.

### 3.4. Functional Enrichment Analysis of Key Targets

**Figure 4:**
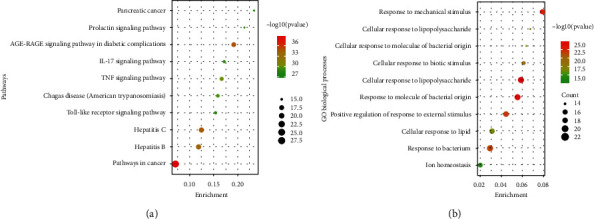
Bubble map of enrichment analysis: (a) KEGG enrichment analysis of Cap against acute lung injury (top 10 were listed); (b) GO enrichment analysis of Cap against acute lung injury (top 10 were listed).

KEGG and GO enrichment analyses were performed to explore the corresponding pathologic processes of Cap key genes in the treatment of acute lung injury. As shown in Figure 4(a), the top 10 signaling pathways were screened and a bubble map was constructed. In particular, the TNF, IL-6, CXCL1, CXCL2, and CXCL10 were mainly enriched in the inflammation pathways, such as the IL-17 and TNF signaling pathway (Supplementary File Table S2). Therefore, these inflammation-related genes were selected and verified in the next animal experiment.

As shown in Figure 4(b), the top 10 GO biological processes were screened and a bubble map was constructed. The results showed that multiple biological processes were involved in acute lung injury treatment, such as response to lipopolysaccharide, response to molecule of bacterial origin, response to mechanical stimulus, positive regulation of response to external stimulus, and response to bacterium, which were closely related to these inflammation-related genes.

Based on the findings of the network pharmacology, we further carried out *in vivo* experiment to validate the therapeutic effect of Cap acting on acute lung injury.

### 3.5. Cap Alleviated LPS-Induced Acute Lung Injury in Neonatal Rats

**Figure 5:**
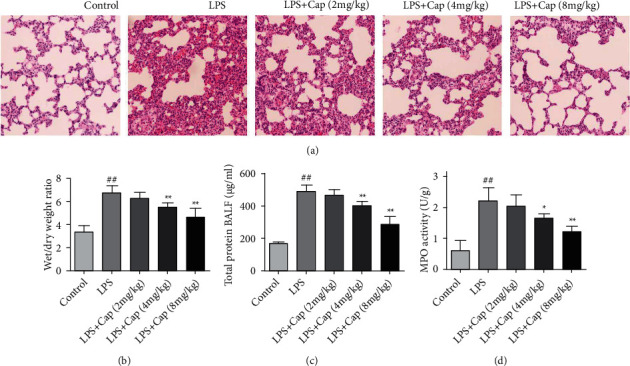
Effects of Cap on experimental pulmonary damage in the acute lung injury animal model. (a) Representative H&E staining (magnification 200x) of lung tissues in each group. (b) The lung wet/dry weight ratio in each group. (c) Total protein levels in BALF. (d) The activity of MPO in lung tissues. Results are expressed as the mean ± standard deviation (*n* = 8). ^##^*P* < 0.01 compared to the control group; ^∗^*P* < 0.05 and ^∗∗^*P* < 0.01 compared to the LPS group.

H&E staining was performed to evaluate the protective effect of Cap. As shown in Figure 5(a), the lung tissue in the control group showed normal pulmonary structures. Conversely, neonatal rats from the model group challenged by LPS exhibited clear and significant histopathology alterations, such as alveolar hemorrhage, inflammatory cell infiltration, and alveolar wall edema. However, these pathological features were alleviated to varying degrees by the treatment with Cap (4 and 8 mg/kg). In particular in the LPS+Cap (8 mg/kg) group, the alveoli were significantly restored in morphology with mild pathological changes. As shown in Figures 5(b)–5(d), the wet/dry weight ratio, total protein of BALF, and MPO activity were significantly increased after LPS administration compared to those in the control group. All those anomalies were reversed to varying degrees by the treatment with Cap (4 and 8 mg/kg), especially in the LPS+Cap group. However, a low dose of Cap (2 mg/kg) does not affect the pulmonary injury in LPS-treated neonatal rats.

### 3.6. Cap Treatment Reduced Inflammatory Cell Accumulation in Neonatal Rats with Acute Lung Injury

**Figure 6:**
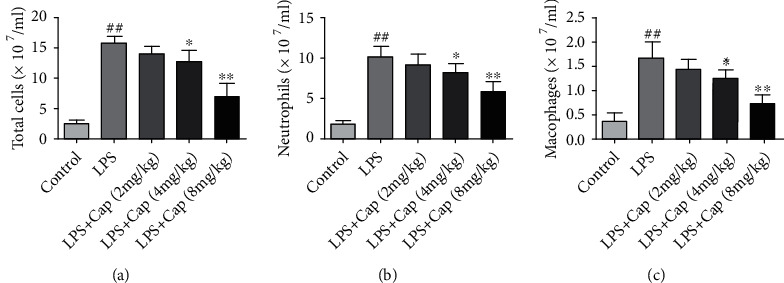
Cap inhibits inflammatory cell accumulation in neonatal rats with acute lung injury. The number of total cells (a), neutrophils (b), and macrophages (c) in BALF. Results are expressed as the mean ± standard deviation (*n* = 8). ^##^*P* < 0.01 compared to the control group; ^∗^*P* < 0.05 and ^∗∗^*P* < 0.01 compared to the LPS group.

LPS stimulation has been reported to induce a strong inflammatory response. In the present study, we measured the levels of inflammatory cell counts in BALF to investigate the underlying protective effects of Cap on lung damage via inhibition of inflammatory accumulation into the alveoli. As shown in Figure 6, the total cells, neutrophils, and macrophages were measured in the control group. Conversely, LPS-exposed neonatal rats showed marked accumulation of total cells, neutrophils, and macrophages, indicating the presence of inflammatory response in the model group. The numbers of infiltrated inflammatory cells were decreased to varying degrees by the treatment with Cap (4 and 8 mg/kg), especially in the LPS+Cap (8 mg/kg) group. However, a low dose of Cap (2 mg/kg) did not decrease this inflammatory cell accumulation in LPS-treated neonatal rats.

### 3.7. Cap Treatment Inhibited the Secretion of Inflammatory Cytokines in Neonatal Rats with Acute Lung Injury

**Figure 7:**
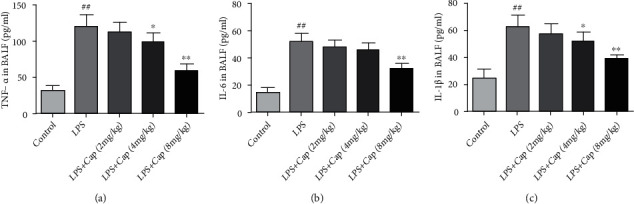
Cap inhibits the release of proinflammatory cytokines in neonatal rats with acute lung injury. The levels of TNF-*α* (a), IL-6 (b), and IL-1*β* (c) in BALF. Results are expressed as the mean ± standard deviation (*n* = 8). ^##^*P* < 0.01 compared to the control group; ^∗^*P* < 0.05 and ^∗∗^*P* < 0.01 compared to the LPS group.

In our study, the proinflammatory cytokines in BALF, including TNF-*α*, IL-6, and IL-1*β*, were measured by using commercial kits to further demonstrate the anti-inflammatory effects of Cap on neonatal rats with acute lung injury. As shown in Figure 7, in the LPS group, the levels of TNF-*α*, IL-6, and IL-1*β* were remarkably higher than those in the control group. The levels of inflammatory cytokines were decreased to varying degrees by the treatment with Cap (4 and 8 mg/kg), especially in the LPS+Cap (8 mg/kg) group. However, a low dose of Cap (2 mg/kg) did not decrease these inflammatory cytokine levels in LPS-treated neonatal rats. These results implied that Cap alleviated LPS-evoked inflammatory response by suppression of inflammatory cell recruitment and decrease in proinflammatory cytokine secretion.

### 3.8. Cap Treatment Improves Antioxidative Enzyme Activities in Neonatal Rats with Acute Lung Injury

**Figure 8:**
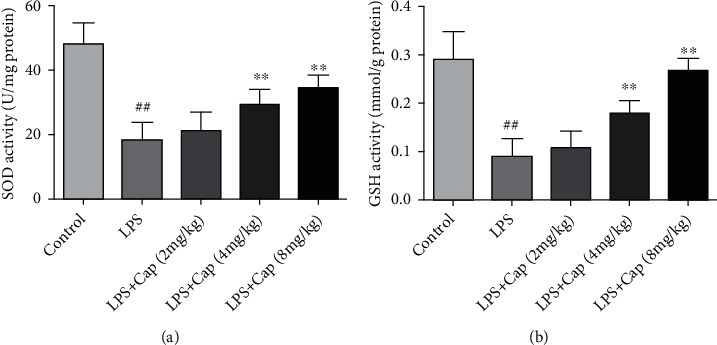
Cap improves the activities of antioxidant enzymes in neonatal rats with acute lung injury. The levels of SOD (a) and GSH (b) in BALF. Results are expressed as the mean ± standard deviation (*n* = 8). ^##^*P* < 0.01 compared to the control group; ^∗∗^*P* < 0.01 compared to the LPS group.

As shown in Figure 8, in the LPS group, the activities of SOD and GSH were remarkably lower than those in the control group. The activities of antioxidative enzymes were improved to varying degrees by the treatment with Cap (4 and 8 mg/kg), especially in the LPS+Cap (8 mg/kg) group. However, a low dose of Cap (2 mg/kg) did not improve antioxidative enzyme activities in LPS-treated neonatal rats. Based on these results, the high dose of Cap (8 mg/kg) was chosen in the next experiment.

### 3.9. Cap Treatment Downregulated Inflammation-Related Gene Expression in Neonatal Rats with Acute Lung Injury

**Figure 9:**
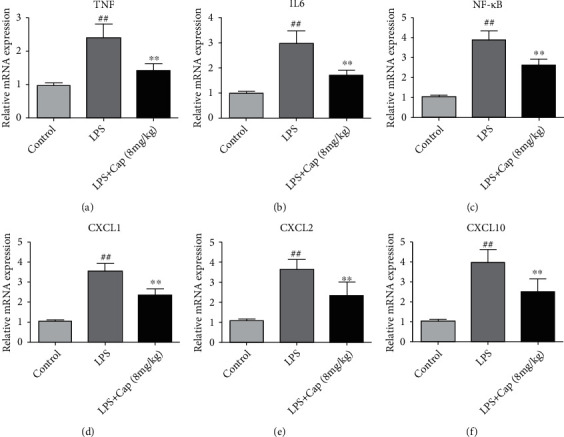
Cap downregulated inflammation-related gene expression in neonatal rats with acute lung injury. The mRNA expression of TNF (a), IL-6 (b), NF-*κ*B (c), CXCL1 (d), CXCL2 (e), and CXCL10 (f) in lung tissue. Results are expressed as the mean ± standard deviation (*n* = 6). ^##^*P* < 0.01 compared to the control group; ^∗^*P* < 0.05 and ^∗∗^*P* < 0.01 compared to the LPS group.

It has been reported that inflammation was involved in the pathogenesis and progression of acute lung injury [21]. In the present study, the candidate Cap targets (TNF, IL-6, CXCL1, CXCL2, and CXCL10) were identified in the network pharmacology analysis further validated by the qRT-PCR experiment. As shown in Figure 9, in the LPS group, the expression of TNF, IL-6, NF-*κ*B, CXCL1, CXCL2, and CXCL10 was remarkably higher than that in the control group. Cap treatment downregulated the expression of TNF, IL-6, NF-*κ*B, CXCL1, CXCL2, and CXCL10 in LPS-treated neonatal rats.

## 4. Discussion

Acute lung injury is an inflammatory disease with a poor prognosis and difficult treatment [22]. The previous report has revealed that acute lung injury pathogenesis is accompanied by functional cell apoptosis, systemic inflammation, and dysfunctions of pulmonary immune homeostasis, which result in endangered life and organ failure [23]. Acute lung injury is a refractory respiratory disease with high mortality and high incidence, which brings a burden to society and families. Although some drugs play an anti-inflammatory effect in acute lung injury, these drugs have not been translated into clinical treatment and their use is currently limited to assist in pulmonary disease management [24, 25]. Therefore, it is necessary to develop novel and effective therapeutic drugs for acute lung injury treatment.

LPS originates from the cell wall of Gram-negative bacteria and induces tissue damage and edema, excessive production of proinflammatory cytokines, and inflammatory cell infiltration [26]. Therefore, LPS-induced acute lung injury is an ideal and reliable animal model for studying pneumonia-related diseases. Cap is an active ingredient of chili pepper, which has attracted increased attention for its various biological activities [15, 16]. Cap exerts the chemopreventive effects against lung carcinogenesis via downregulating the expression of inflammatory cytokines [17]. Thus, we speculated that Cap may also have a therapeutic effect on inflammatory pulmonary disease. In the present study, network pharmacology and animal experiment were employed to investigate the therapeutic effect of Cap on acute lung injury.

First, we used the network pharmacology method to screen the potential targets and signaling pathways of Cap in the treatment of acute lung injury. The results of the PPI network revealed that 619 potential targets of Cap have a vital role in acute lung injury treatment, and some inflammatory-related genes, such as CXCL8, CXCR4, CXCL1, CXCL2, IL-6, CXCL10, CCL20, CCL19, IL-2, and TNF, were screened out to be closely related to Cap on acute lung injury treatment. Besides, the KEGG enrichment analysis revealed that IL-17 and TNF signaling pathways were the important pathways of Cap against acute lung injury. Next, an animal experiment further confirmed that Cap alleviates lung damage via inhibiting the proinflammatory generation in neonatal rats with acute lung injury.

One of the pathogeneses of acute lung injury is that the imbalance of inflammatory response deteriorates the damage of endothelial or epithelial cells [27, 28], which causes an increase in protein levels of the alveoli [29]. Histologically, acute lung injury is characterized by the formation of pulmonary fibrosis, increased alveolar-capillary permeability, a mass of apoptosis in alveolar epithelial cells, and severe acute inflammatory response [30]. In addition, the release of proinflammatory cytokines, excessive neutrophil migration, and destruction of alveolar-capillary membrane integrity are also the major manifestations of acute lung injury cytopathology [31–33]. It has been demonstrated that neutrophils and macrophages are the major sources of multiple inflammatory cytokines in the occurrence and development of lung injury. In response to LPS stimulation, a large number of neutrophils accumulate in the pulmonary capillaries where they are activated, releasing cytotoxic constituents and resulting in damage of pulmonary cells [34, 35]. A previous report revealed that T lymphocytes and neutrophils promote the development of pulmonary disease [36]. MPO, a proinflammatory enzyme, is mainly generated by activated neutrophils and it could promote inflammation to worsen and prolong. Previous studies have demonstrated that the MPO activity of the pulmonary tissue is often considered a biomarker of neutrophil infiltration [37]. In the present study, the histopathological changes of pulmonary tissue induced by LPS were well recovered by Cap treatment. The pulmonary edema and MPO activity of neonatal rats with acute lung injury were also alleviated. Besides, the pulmonary inflammation was ameliorated by Cap, as evidenced by decreasing neutrophils and macrophages and inhibiting the secretion of inflammatory cytokines.

A variety of inflammatory mediators are overexpressed in the lung tissue of the acute lung injury model, and TNF and IL-17 signaling pathways were involved in the release of proinflammatory cytokines [11, 38]. TNF-*α*, a primary proinflammatory mediator in organisms, promotes the activation of macrophages and pulmonary endothelial cells and evokes inflammatory responses [39]. IL-6 is one of the proinflammatory mediators released during acute lung injury and is mainly generated by the innate immune system. Previous reports have revealed that pulmonary-derived IL-6 induces idiopathic pneumonia syndrome through the promotion of Th17 differentiation [40]. In response to LPS stimulation, the generation of chemoattractants CXCL1/CXCL2 initiates an early phase of neutrophil recruitment [41]. Neutrophils were recruited by CXCL1 into infected tissue to eliminate pathogens in acute lung injury [42]. CXCL2 was mostly overexpressed on resident alveolar macrophages during LPS-induced lung damage [43]. The gene CXCL10 was identified as a potential biomarker for the diagnosis and treatment of acute respiratory distress syndrome [44]. NF-*κ*B, an upstream transcription factor, evokes lots of genes involved in inflammation, cell adhesion, and immune regulation [45]. The previous report demonstrated that NF-*κ*B activation is implicated in the LPS-induced acute lung injury [46]. Besides, oxidative stress is reconsidered as another inducer that is implicated in the progression of lung injury [47]. It has been reported that LPS treatment evokes the generation of reactive oxygen species and inflammatory cytokines, thus causing acute lung injury in a mouse model [48]. Thus, any ingredient that restrains inflammation and oxidative stress might potentially exert therapeutic effects against lung injury. In this experiment, Cap treatment prevented the secretion of proinflammatory cytokines, improved the activities of antioxidant enzymes, and inhibited the upregulation of TNF, IL-6, NF-*κ*B, CXCL1, CXCL2, and CXCL10 induced by LPS, which demonstrates that the TNF and IL-17 signaling pathways are important for acute lung injury treatment with Cap. Thus, these results demonstrated that Cap effectively blocked the pulmonary inflammation and oxidative stress to control acute lung injury progression and verified the results of network pharmacology.

## 5. Conclusions

In conclusion, the present study preliminarily predicted the potential genes and signaling pathways of Cap alleviating acute lung injury via network pharmacology. Meanwhile, we performed an animal experiment that confirmed the results of network pharmacology. These results provide a scientific basis for the prevention and treatment of acute lung injury.

## Data Availability

The data used to support the study are available from the corresponding author.

## Supplementary Materials

Supplementary Materials Table S1: sequences of primers used in quantitative real-time PCR. Table S2: results of KEGG enrichment analysis.
