# Supplementary material for: Integrated Network Pharmacology and Experimental Validation Approach to Investigate the Therapeutic Effects of Capsaicin on Lipopolysaccharide-Induced Acute Lung Injury
Source: Mediators Inflamm. 2022 Jan 30;2022:9272896. doi: 10.1155/2022/9272896 (PMC8818435; doi:10.1155/2022/9272896)
Supplement: Supplementary Materials — Table S1: sequences of primers used in quantitative real-time PCR. Table S2: results of KEGG enrichment analysis. [file 9272896.f1.pdf]

Table S1. Sequences of primers used quantitative real-time PCR.

Gene	Forward primer (5' to 3')	Reverse primer (5' to 3')
NF-κB	GCAACTCTGTCCTGCACCTA	CTGCTCCTGAGCGTTGACTT
CXCL1	GCTTGAAGGTGTTGCCCTCAG	AGAAGC CAGCGTTCACCAGAC
IL6	TCCTACCCCAACTTCCAATGCTC	TTGGATGGTCTTGGTCCTTAGCC
TNF	ATGGCATGGATCTCAAAGAC	CGGACTCCGTGATGTCTAAG
CXCL2	TCCTCAATGCTGTACTGGTCC	ATGTTCTTCCTTTCCAGGTC
CXCL10	TCTCTCCACCTCCCTTTACCC	CTTGTCCATCACGCTGTAGT
GAPDH	CTGACGAAGGACAATGAGTGCACAGCGC	ATTCCACATCACAAGACTTCGCTCAGCC

Table S2. Results of KEGG enrichment analysis.

Pathways	enrichment	pvalue	count	Genes
Pathways in cancer	0.07088608	3.32866E-39	28	AGTR1, AKT1, BDKRB1, BDKRB2, CTNNB1, EGF, EGFR, FOS, HRAS, HSP90AA1, IGF1, IL6, CXCL8, JAK1, JUN, NFKB1, PIK3CA, PIK3R1, MAPK1, MAPK3, MAPK8, RELA, RXRA, STAT1, STAT3, TP53, VEGFA, CXCR4
AGE-RAGE signaling pathway in diabetic complications	0.19191919	1.05058E-34	49	AGTR1, AKT1, MAPK14, EDN1, HRAS, IL6, CXCL8, JUN, NFKB1, PIK3CA, PIK3R1, MAPK1, MAPK3, MAPK8, RELA, STAT1, STAT3, TNF, VEGFA, CTNNB1, EGF, EGFR, CXCL10, JAK1, RXRA, TP53, FOS, SRC, STAT6, IL2, CXCL1, CXCL2, CCL20, BDKRB2, KNG1, HSP90AA1, POMC, ESR1, BDKRB1, CNR1, OPRM1, CXCR4, IGF1, F2, CCL19, CHRM2, GCG, NPY, SST
Hepatitis C	0.12426036	3.53551E-34	21	AKT1, MAPK14, CTNNB1, EGF, EGFR, HRAS, CXCL8, CXCL10, JAK1, NFKB1, PIK3CA, PIK3R1, MAPK1, MAPK3, MAPK8, RELA, RXRA, STAT1, STAT3, TNF, TP53

Hepatitis B	0.11864407	9.84786E-34	21	AKT1, MAPK14, FOS, HRAS, IL6, CXCL8, JAK1, JUN, NFKB1, PIK3CA, PIK3R1, MAPK1, MAPK3, MAPK8, RELA, SRC, STAT1, STAT3, STAT6, TNF, TP53
TNF signaling pathway	0.16666667	1.18443E-31	18	AKT1, MAPK14, EDN1, FOS, CXCL1, CXCL2, IL6, CXCL10, JUN, NFKB1, PIK3CA, PIK3R1, MAPK1, MAPK3, MAPK8, RELA, CCL20, TNF
Chagas disease (American trypanosomiasis)	0.1588785	1.67894E-29	17	AKT1, BDKRB2, MAPK14, FOS, IL2, IL6, CXCL8, JUN, KNG1, NFKB1, PIK3CA, PIK3R1, MAPK1, MAPK3, MAPK8, RELA, TNF
Pancreatic cancer	0.234375	8.01573E-29	15	AKT1, EGF, EGFR, JAK1, NFKB1, PIK3CA, PIK3R1, MAPK1, MAPK3, MAPK8, RELA, STAT1, STAT3, TP53, VEGFA
IL-17 signaling pathway	0.17204301	2.30815E-28	16	MAPK14, FOS, CXCL1, CXCL2, HSP90AA1, IL6, CXCL8, CXCL10, JUN, NFKB1, MAPK1, MAPK3, MAPK8, RELA, CCL20, TNF
Prolactin signaling pathway	0.21428571	3.60007E-28	15	AKT1, MAPK14, ESR1, FOS, HRAS, NFKB1, PIK3CA, PIK3R1, MAPK1, MAPK3, MAPK8, RELA, SRC, STAT1, STAT3
Toll-like receptor signaling pathway	0.15384615	1.58846E-27	16	AKT1, MAPK14, FOS, IL6, CXCL8, CXCL10, JUN, NFKB1, PIK3CA, PIK3R1, MAPK1, MAPK3, MAPK8, RELA, STAT1, TNF
